# Evolution of Growth Habit, Inflorescence Architecture, Flower Size, and Fruit Type in Rubiaceae: Its Ecological and Evolutionary Implications

**DOI:** 10.1371/journal.pone.0040851

**Published:** 2012-07-16

**Authors:** Sylvain G. Razafimandimbison, Stefan Ekman, Timothy D. McDowell, Birgitta Bremer

**Affiliations:** 1 Bergius Foundation, The Royal Swedish Academy of Sciences and Botany Department, Stockholm University, Stockholm, Sweden; 2 Museum of Evolution, Uppsala University, Uppsala, Sweden; 3 Department of Biological Sciences, East Tennessee State University, Johnson City, Tennessee, United States of America; University of Massachusetts, United States of America

## Abstract

During angiosperm evolution, innovations in vegetative and reproductive organs have resulted in tremendous morphological diversity, which has played a crucial role in the ecological success of flowering plants. Morindeae (Rubiaceae) display considerable diversity in growth form, inflorescence architecture, flower size, and fruit type. Lianescent habit, head inflorescence, small flower, and multiple fruit are the predominant states, but arborescent habit, non-headed inflorescence, large flower, and simple fruit states occur in various genera. This makes Morindeae an ideal model for exploring the evolutionary appearances and transitions between the states of these characters. We reconstructed ancestral states for these four traits using a Bayesian approach and combined nuclear/chloroplast data for 61 Morindeae species. The aim was to test three hypotheses: 1) self-supporting habit is generally ancestral in clades comprising both lianescent and arborescent species; 2) changes from lianescent to arborescent habit are uncommon due to “a high degree of specialization and developmental burden”; 3) head inflorescences and multiple fruits in Morindeae evolved from non-headed inflorescences and simple fruits, respectively. Lianescent habit, head inflorescence, large flower, and multiple fruit are inferred for Morindeae, making arborescent habit, non-headed inflorescence, small flower, and simple fruit derived within the tribe. The rate of change from lianescent to arborescent habit is much higher than the reverse change. Therefore, evolutionary changes between lianescent and arborescent forms can be reversible, and their frequency and trends vary between groups. Moreover, these changes are partly attributed to a scarcity of host trees for climbing plants in more open habitats. Changes from large to small flowers might have been driven by shifts to pollinators with progressively shorter proboscis, which are associated with shifts in breeding systems towards dioecy. A single origin of dioecy from hermaphroditism is supported. Finally, we report evolutionary changes from headed to non-headed inflorescences and multiple to simple fruits.

## Introduction

During angiosperm evolution, changes in vegetative and reproductive organs have resulted in remarkable morphological diversity, which has played an important role in the ecological success of flowering plants [1]. The fusion of clustered fruits into multiple fruits (or syncarps) has occurred repeatedly in different lineages [1]–[2]. Some multiple fruits are important food sources for a wide range of animals, and the evolution of this type of compound fruit has been hypothesized as a result of selection by large animals [3]. This is based on the fact that multiple fruits are generally favored and their seeds are effectively dispersed by large frugivorous dispersers [4]. Flowering plants from different groups produce edible multiple fruits that are economically important. Examples include jackfruits and breadfruits (Moraceae), pineapples (Bromeliaceae), and noni fruits (Rubiaceae). Despite their crucial roles in different ecosystems and for the human society, little is known about the evolution of multiple fruits. This is partly due to the lack of robust phylogenies for the lineages that contain species producing multiple fruits and species bearing simple fruits. Molecular-based phylogenies are essential for placing patterns of any heritable trait in an evolutionary context [5].

**Figure 1:**
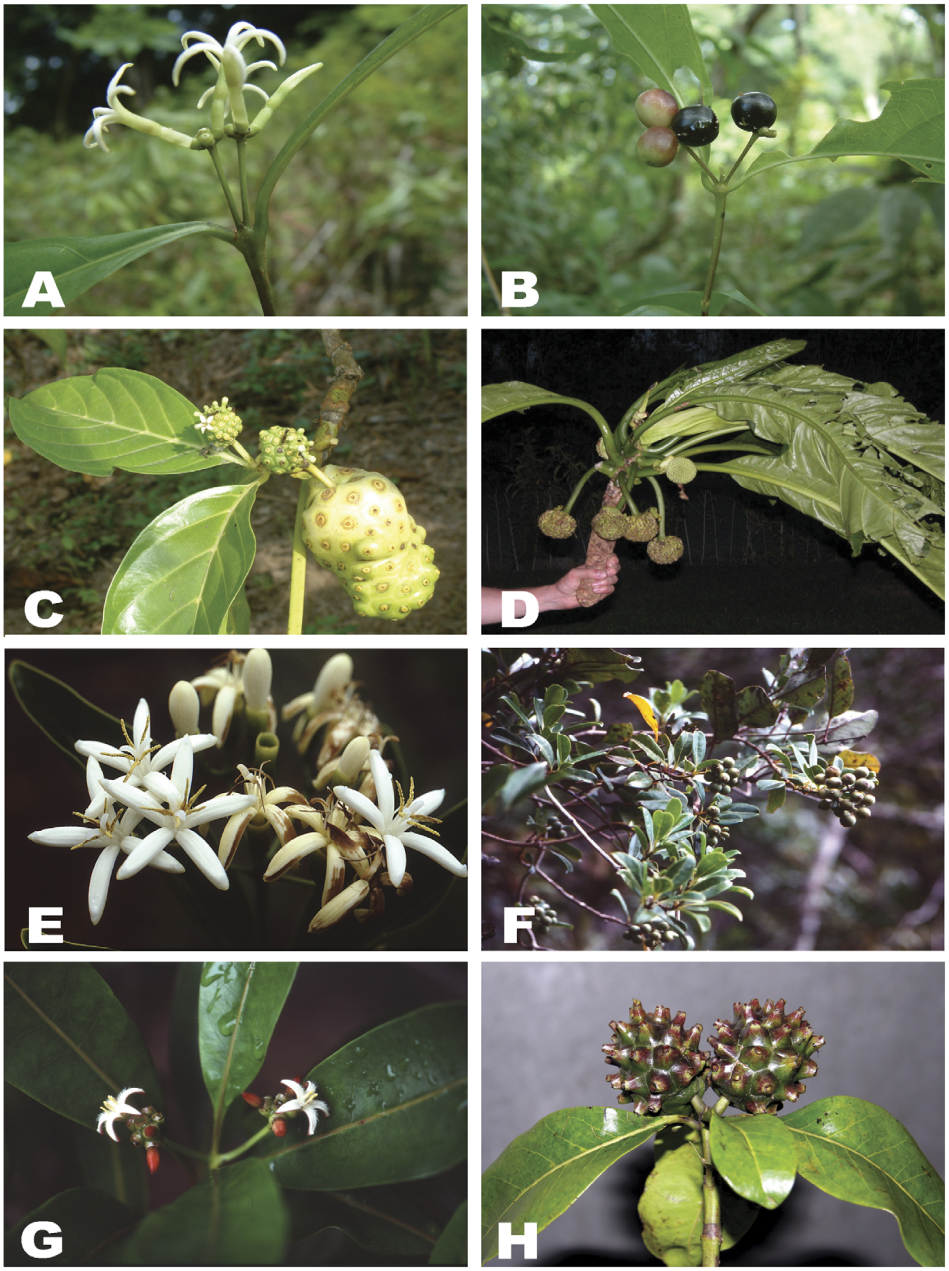
Characteristics and morphological variation of the tribe Morindeae (for details see text). A–B: *Appunia debilis*; C: *Morinda citrifolia*; D: *Morinda pacifica*; E: *Coelospermum fragrans*; F: *Coelospermum balansanum*; G: *Gynochthodes kanalensis*; and H: *Gynochthodes retusa* (A–C by T. D. McDowell; D by F. Tronquet; E–G by J. T. Johansson; and H by K. Kainulainen).

In the coffee family (Rubiaceae), most taxa with multiple fruits are members of the tribes Naucleeae [6]–[7] and Morindeae [8]–[10]. Fruits in Morindeae, belonging to the Psychotrieae alliance in the subfamily Rubioideae, are predominantly multiple fruits composed of two to many fully to basally fused drupaceous (fleshy) fruits (Fig. 1C–D, H), which are derived from ovaries of the adjacent flowers. This type of compound fruit is found in three (*Coelospermum* Blume, *Gynochthodes* Blume (Fig. 1H), and *Morinda* L. (Fig. 1C)) of the five genera currently recognized in the tribe, and is absent in the other two genera (*Appunia* Hook.f. and *Siphonandrium* K.Schum.) the infructescences (fruiting stage of inflorescences) of which are formed by clusters of simple, drupaceous fruits (Fig. 1B). A few members of *Coelospermum* are characterized by branched or headed infructescences bearing pedicellate (stalked), drupaceous fruits (Fig. 1F), while some *Gynochthodes* species have infructescences composed of pedicellate, drupaceous fruits grouped in umbels (flat-topped or rounded flower/fruit clusters with the pedicels arising from more or less the same point) or fascicles (tight bundles). It has been postulated by McClatchey [11] that multiple fruits of the broadly circumscribed *Morinda* (*Morinda* sensu lato), which included all lianescent and arborescent *Morinda* species with multiple fruits recently transferred to *Gynochthodes* [8], evolved from an ancestor with umbels and simple fruits by suppression of the pedicels and fusion of the ovaries of the adjacent flowers. This would imply that multiple fruits of *Morinda* and *Gynochthodes* (both sensu Razafimandimbison et al. [8]–[9]) are derived in Morindeae.

Besides its fruit diversity, Morindeae are also diverse in growth form, inflorescence architecture, and flower size. Lianescent (climbing) habit, headed inflorescence, and small flower are the predominant states, and occur in different genera of the tribe. This makes Morindeae an attractive model for exploring the evolutionary appearances and transitions between the major states of the four major characters (i.e., growth habit, inflorescence architecture, flower size, and fruit type) from a phylogenetic perspective. The recently published molecular phylogeny of Morindeae [9] provides a solid basis for such a study. These traits have been revealed to be evolutionarily labile in Morindeae [9], however a proper ancestral state reconstruction (hereafter called ASR) is essential in order to better understand their evolution. Moreover, a combination of these four characters has been used for circumscribing the five recognized genera of the tribe (Table 1).

**Table 1:** Morphological characteristics and other important information of the five recognized genera of the tribe Morindeae.

	*Appunia* Hook.f.	*Coelospermum* Blume	*Gynochthodes* sensulato Blume	*Morinda* sensustricto L.^1^	*Siphonandrium*K.Schum.^2^
Geographic distribution	Neotropics	Tropical Asia andAustralasia	Tropical Asia, Australasia, andMadagascar	Pantropical	New Guinea
Number of species	Ca. 12	Ca. 11	Ca. 95	Ca. 40	1
Growth habit	Mostly frutescent	Mostly woody lianescent	Mostly woody lianescent,	Mostly arborescent and frutescent	Lianescent
Inflorescence architecture	Head inflorescences	Mostly non-headed inflorescences	Mostly head inflorescences	Head inflorescences	Head inflorescences
Flower size	Large (corolla tubes5–10 (23–24) mm long> corolla lobes 0.5–7 (15–17) mm) long	Small (corolla tubes3–7 (11) mm long <corolla lobes 4.5–16 mm) long	Small (corolla tubes0.7–5.5 mm long <corolla lobes 1.5–11 mm) long	Large (corolla tubes5–40 (80) mm long >corolla lobes 1–14 (22) mm) long	Small (corolla tubesca. 3 mm long >corolla lobes ca. 5 mm long)
Breeding systems	Hermaphroditic	Androdioecious or dioecious or functionally dioecious	Androdioecious or dioecious orfunctionally dioecious	Hermaphroditic	Dioecious
Fruit type	Simple, drupaceous fruits	Mostly simple, drupaceous fruits	Mostly multiple fruits	Multiple fruits	Simple, drupaceous fruits

1 All lianescent, dioecious species of *Morinda* with small flowers have recently been transferred to *Gynochthodes* [8].

2 Filaments of *Siphonandrium* are tightly fused and its anthers are glued together, all forming a staminal tube. This feature is unique within Morindeae.

Morindeae comprise ca. 100 woody climbing species (ca. 62%), with only ca. 54 arborescent (tree or tree-like) and frutescent (shrubby) species (ca. 28%), and two suffrutescent (shrubby plants having woody stems only at the base) species. Frutescent plants are common in *Appunia*. Both arborescent and frutescent plants are predominant in *Morinda* but are extremely rare in *Coelospermum* (the arborescent *C. reticulatum* (F.Muell.) Benth.) and *Gynochthodes* (frutescent *G. decipiens* (Schltr.) Razafim. & B.Bremer and the arborescent *G. trimera* (Hillebr.) Razafim. & B.Bremer). Conversely, lianescent plants (woody vines or climbers) in the tribe are found mostly in *Gynochthodes* [8]–[9], with only one species in *Appunia* (*A. megalantha* C.M.Taylor & Lorence, [12]), two species in *Morinda* (*M. longiflora* G.Don and *M. morindoides* (Baker) Milne-Redh., [9]), and 10 species in *Coelospermum* [13]. There are at least two different (but not mutually exclusive) hypotheses regarding the evolutionary transitions between lianescent and arborescent growth forms. The first hypothesis considers self-supporting (arborescent or frutescent) habit to be the common ancestral condition for clades containing both lianescent and arborescent/frutescent species [14]–[15]. The second hypothesis states that the evolutionary change from lianescent to arborescent/frutescent habit is uncommon, because “the evolution of lianescence can carry a high degree of specialization and developmental burden that might limit evolution back to self-supporting growth forms” [16].

Head inflorescences (or heads) occur when two or more flowers are borne on a common receptacle (the end of the inflorescence stalk upon which the floral organs are borne). In Morindeae, these heads are composed of two to 50 flowers that are clustered tightly on the receptacles (Fig. 1A, C–D, G). These inflorescences may contain a single head or two to several heads, which are in turn arranged into various branching forms: umbels, corymbs (flat-topped or round-topped, racemose inflorescences with the lower pedicels longer than the upper), or panicles (branched clusters of flowers). Within a head the ovaries of the adjacent flowers may be fused or free. The majority of Morindeae species with heads have fused ovaries, but in *Appunia* (Fig. 1A) and *Siphonandrium* the ovaries are free. It is important to note that the degree of the fusion of ovaries prior to fruit development (pre-genital fusion) varies greatly among species, from only basally to completely fused. Only 27 Morindeae species (nine from *Coelospermum* and 18 from *Gynochthodes*) bear non-headed inflorescences, which are arranged in umbels, or compound umbels, or fascicles (in the latter genus) and panicles or corymbs (in the former genus). We postulated that heads evolved from non-headed inflorescences in Morindeae. In addition, inflorescences in Morindeae are mostly terminal on the shoot, sometimes leaf-opposed ("pseudo-terminal") and axillary. Axillary inflorescences are found in *Siphonandrium*, at least three species of *Appunia*, and ca. 18 species of *Gynochthodes*. Leaf-opposed inflorescences distinguish the mostly Asian, arborescent *Morinda* clade (including the Neotropical *M. royoc* L., the pantropical *M. citrifolia* L., and the African *M. chrysorhiza* (Thonn.) DC. and *M. lucida* Benth.) from the remaining *Morinda* [9]; this type of inflorescence is also known to occur in *Appunia* (e.g., *A. surinamensis* (Bremek.) Steyerm.).

In Morindeae, flowers in the same inflorescence or head appear to open successively over days or weeks [17] (Fig. 1A, C, E, G). The flowers vary greatly in size (Table 1); large flowers (corolla tube length/corolla lobe length >1) are found in *Appunia* and *Morinda* (Fig. 1A, C) and presumably pollinated by larger insects, such as long-proboscis moths [18]. Plants with small flowers (corolla tube length/corolla lobe length <1) are restricted to *Coelospermum*, *Gynochthodes*, and *Siphonandrium* (Fig. 1E, G), and are most likely to be pollinated by small insects (e.g., short-proboscis moths or small bees or flies) [19]–[20]. Overall, the species of *Morinda* have larger flowers than the species of *Appunia*, with the exception of the lianescent *A. megalantha* (corolla tubes of 23–24 mm long > corolla lobes of 15–17 mm long, [12]). Within *Morinda*, seven African species have much larger flowers than the remaining species of the genus. The flowers of *Coelospermum* are larger than those of the species of *Gynochthodes* [9]. Furthermore, Morindeae species vary in their breeding systems, and flowers are either bisexual or unisexual or functionally unisexual. Only the hermaphroditic condition has been reported in *Appunia* and *Morinda* [21], while the androdioecious (male and hermaphroditic individuals) [21]–[22], strict or functional dioecious [23]–[24], and hermaphroditic [21], [24] conditions are all known in *Gynochthodes* and *Coelospermum* [21]–[24]. The New Guinean *Siphonandrium* is dioecious (Table 1).

We reconstructed the evolution of fruit type, inflorescence architecture, flower size, and growth form across a phylogeny of the tribe Morindeae. We were particularly interested in testing the following hypotheses: 1) self-supporting habit is generally plesiomorphic in clades comprising both lianescent and arborescent species [14]; 2) evolutionary changes from lianescent to arborescent/frutescent habit are less frequent than the reverse change, from arborescent/frutescent to lianescent habit [16]; 3) and Head inflorescences and multiple fruits in Morindeae evolved from non-headed inflorescences and simple fruits, respectively. The ecological and evolutionary implications of the findings of this study are discussed.

## Results

**Figure 2:**
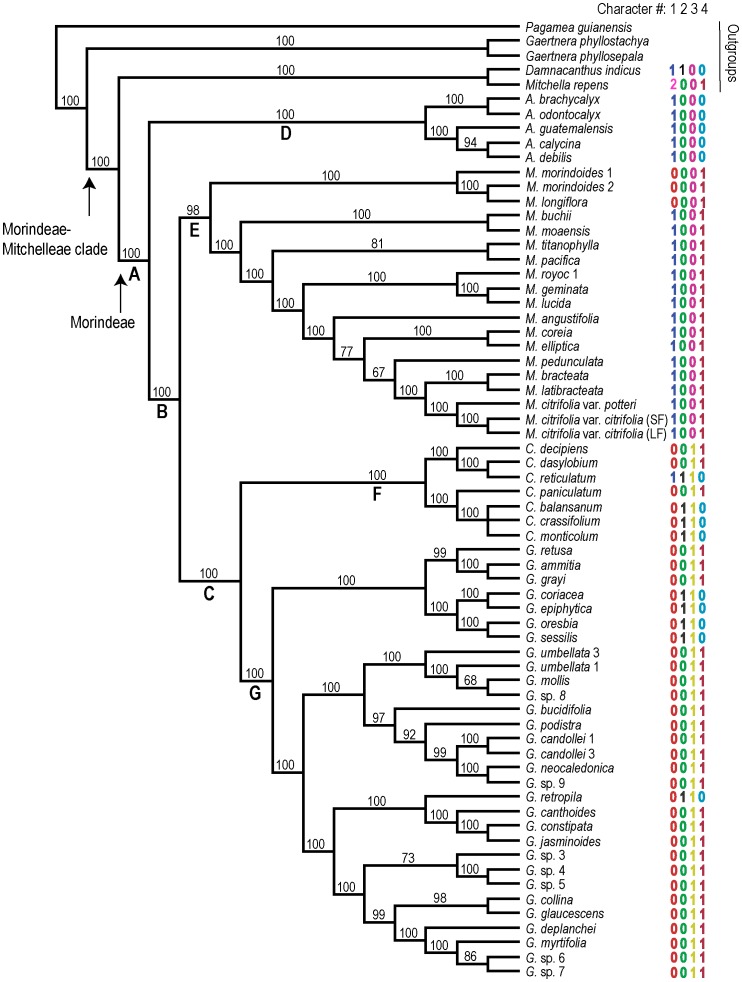
Bayesian majority rule consensus tree from the combined nrETS/nrITS/*trn*T-F data of 61 Morindeae taxa. Values above nodes are the posterior probabilities. Capital letters A–G denote selected nodes whose state probabilities were estimated for the states of the four characters (1–4). Data shown across the tips are growth habit (character 1: 0 =  lianescent, 1 =  arborescent, 2 =  herbaceous), inflorescence architecture (character 2: 0 =  headed inflorescences (heads); 1 =  non-headed inflorescences), flower size (character 3: 0 =  large, 1 =  small), and fruit type (character 4: 0 =  simple fruits, 1 =  fused or multiple fruits). SF and LF stand for small and large fruits, respectively.

The Bayesian majority rule consensus tree generated from the combined nrETS/nrITS/*trn*T-F data and shown in Figure 2 was fully resolved. Its overall topology is almost identical to that of the Bayesian majority rule consensus tree published in Razafimandimbison et al. [9].

### Ancestral State Reconstructions of Growth form, Inflorescence Architecture, Flower Size, and Fruit Type in Morindeae

Two types of ASRs were performed (one with the outgroup *Damnacanthus indicus* C.F.Gaertn. and *Mitchella repens* L. (tribe Mitchelleae), hereafter called ASR with outgroup, and the other without the outgroup, hereafter called ASR without outgroup), to infer the ancestral states of growth form, inflorescence architecture, flower size, and fruit type (characters 1–4, respectively) at seven important nodes of Morindeae (nodes A–G, Fig. 2). The results of these ASRs are summarized in Tables 2,3. For growth form (character 1) and flower size (character 3) the ratios q_01_/q_10_ from the ASRs with outgroup, respectively, were 4.820 and 2.691 for Morindeae (Table 2). This indicates that the rates of changes from lianescent to arborescent habit and from large to small flower were higher than the rates of the reverse directions, from arborescent to lianescent habit and from small to large flower. In contrast, for inflorescence architecture (character 2) and fruit type (character 4), the ratios q_01_/q_10_, respectively, were 0.676 and 0.677 for Morindeae (Table 2); this means that the rates of changes from non-headed to headed inflorescence and from multiple to simple fruit are higher than the rates of the reverse changes, from headed to non-headed inflorescence and from simple to multiple fruit.

**Table 2:** Bayesian reconstruction of ancestral states in the four characters (1–4) at seven nodes (A–G) across a posterior sample of trees including Morindeae but no outgroup.

	Character*			
	1 (growth form)	2 (inflorescence architecture)	3 (flower size)	4 (fruit type)
Coded character states	lianescent = 0 and arborescent = 1	headed = 0 andnon-headed = 1	large = 0 andsmall = 1	simple = 0 andfused = 1
q_01_/q_10_	4.820	0.676	2.691	0.677
κ	0.961 (0.363–1.508)	0.711 (0.111–1.315)	1.124 (0.239–1.950)	0.787 (0.142–1.332)
Node A (Morindeae)	0.861	0.584	0.980	0.370
Node B (the *Morinda-Coelospermum-Gynochthodes* clade)	0.992	0.586	0.878	0.181
Node C (the *Coelospermum-Gynochthodes*)	0.998	0.584	0.000	0.185
Node D (*Appunia*)	0.007	0.909	1.000	0.989
Node E (*Morinda*)	0.860	0.910	1.000	0.025
Node F (*Coelospermum*)	0.998	0.565	0.000	0.214
Node G (*Gynochthodes*)	1.000	0.583	0.000	0.186

* For each character 1–4, the following information is provided: the ratio of the average rate q_01_ to the average rate q_10_, the average and 95% highest posterior density (HPD) of κ, and the marginal posterior probabilities of having state 0 in each of the seven nodes (A–G). As all characters are binary, the marginal posterior probability of having state 1 is one minus the probability of state 0. The 95% HPD of κ excludes 0 in all cases, which is a strong indication that branch lengths carry information about the amount of change in the morphological characters.

**Table 3:** Bayesian reconstruction of ancestral states in the four characters (1–4) at seven nodes (A–G) across a posterior sample of trees including Morindeae as well as the outgroup taxa *Damnacanthus indicus* and *Mitchella repens* (Mitchelleae).

	Character			
	1 (growth form)	2 (inflorescence architecture)	3 (flower size)	4 (fruit type)
Coded character states	lianescent (0), arborescent (1),and herbaceous (2)	headed (0) and non-headed (1)	large (0) and small (1)	simple (0) and fused (1)
κ	0.981 (0.394–1.516)	0.671 (0.061–1.259)	1.144 (0.221–1.996)	0.784 (0.128–1.312)
Node of Morindeae-Mitchelleae clade	0.258, 0.216, 0.524	0.513	0.996	0.359
Node A (Morindeae)	0.733, 0.199, 0.067	0.566	0.994	0.371
Node B (the *Morinda-Coelospermum-* *Gynochthodes* clade)	0.970, 0.011, 0.019	0.568	0.886	0.166
Node C (the *Coelospermum-Gynochthodes*)	0.991, 0.001, 0.008	0.565	0.000	0.171
Node D (*Appunia*)	0.007, 0.980, 0.013	0.916	1.000	0.987
Node E (*Morinda*)	0.733, 0.202, 0.065	0.917	1.000	0.023
Node F (*Coelospermum*)	0.990, 0.001, 0.009	0.540	0.000	0.207
Node G (*Gynochthodes*)	0.998, 0.000, 0.002	0.564	0.000	0.172

* For each character 1–4, the average and 95% highest posterior density of κ is provided. For character 1, we also provide the marginal posterior probabilities of having state 0, 1, and 2, respectively, in each of the eight selected nodes (the Mitchelleae-Morindeae root node and nodes A–G). For character 2–4, we provide the marginal posterior probabilities of having state 0 for the same eight nodes. The 95% HPD of κ excludes 0 in all cases, which is a strong indication that branch lengths carry information about the amount of change in the morphological characters.

The node of the Morindeae-Mitchelleae clade ( =  the Mitchelleae-Morindeae common ancestor) (Fig. 2) was inferred with strong and moderate support, respectively, as large flower and multiple fruit in the ASR with outgroup; however, the results of this analysis were inconclusive for growth habit and inflorescence architecture (Table 3). The outcomes of the ASRs with and without outgroup were very similar for the seven important nodes of Morindeae (nodes A–G, Fig.2) (Tables 2,3). At node A (the Morindeae common ancestor) the lianescent habit, head inflorescence, large flower, and multiple fruit states were inferred; however the support was weak for head inflorescence, moderate for lianescent habit and multiple fruit, and strong for large flower. Within Morindeae lianescent habit was strongly inferred at node B (the *Morinda-Coelospermum-Gynochthodes* clade), node C (*Coelospermum-Gynochthodes* clade), node E (*Morinda*), node F (*Coelospermum*), and node G (*Gynochthodes*), while arborescent habit was resolved at node D (*Appunia*). For inflorescence architecture (character 2) head inflorescence was inferred at nodes B-G; the support was moderate for nodes B–C and F–G but strong for nodes D–E. For flower size (character 3) large flower was strongly inferred at nodes B and D–E, whereas nodes C and F–G were unambiguously resolved as small flower. Finally, for fruit type (character 4) fused fruit (multiple fruit) was strongly inferred at nodes B–C and nodes E–G, however simple fruit was highly resolved at node D (Tables 2,3).

## Discussion

We performed ASRs with and without outgroup in order to assess the influence of the outgroup taxa on the outcomes. The fact that the results of these two ASRs are very similar for the seven nodes of Morindeae (nodes A–G, Fig. 2, Tables 2,3) suggests that the inclusion of the outgroup taxa (*Damnacanthus indicus* and *Mitchella repens*) has almost no effect on the analyses. The ASR with outgroup infers large flower with strong support and multiple fruit with moderate support at the node of the Morindeae-Mitchelleae clade. Lianescent habit, head inflorescence, large flower, and multiple fruit are inferred at node A (Morindeae) (Tables 2,3). If these inferences are correct, these states are interpreted as plesiomorphic for Morindeae and are plesiomorphic within Morindeae, with respect to the later changes (i.e., arborescent habit, non-headed inflorescence, small flower, and simple fruit) in the group.

### Evolution of Growth form in Morindeae and its Ecological and Evolutionary Implications

Two-thirds of the species in Morindeae are represented by the lianescent species of *Gynochthodes*, while only two *Morinda* and one *Appunia* species are lianas. Conversely, a single species of *Coelospermum* (*C. reticulatum*) and two *Gynochthodes* (*G. decipiens* and *G. trimera*, not investigated in this study) species are arborescent. The fact that lianescent habit is inferred at node A (Morindeae) means that this state is plesiomorphic at node B (the *Morinda-Coelospermum-Gynochthodes* clade), node C (the *Coelospermum-Gynochthodes* clade), and nodes E–G (*Morinda*, *Coelospermum*, and *Gynochthodes*, respectively). Arborescent habit is inferred as apomorphic at node D (*Appunia*), and seems to have arisen at least three times within Morindeae: *Appunia*, *Morinda* (node E: *M. butchii* Urb. to *M. citrifolia* L., Fig. 2), and *Coelospermum* (node F: *C. reticulatum*, Fig. 2). Our findings provide no support for the reported prevalence of a plesiomorphic arborescent habit in lineages containing both lianescent and arborescent plants [14]. In fact, a plesiomorphic lianescent habit and multiple independent origins of arborescent from lianescent habit have recently been inferred for the primarily lianescent subfamily Secamonoideae in the family Apocynaceae [16] and the family Menispermaceae [25]. Moreover, the rate of change from lianescent to arborescent habit in Morindeae is significantly higher than the reverse change, from arborescent to lianescent habit (q_01_/q_10_ = 4.820>1, Table 2); this is inconsistent with Lahaye et al.’s [16] claim that “the evolution of lianescence can carry a high degree of specialization and developmental burden that might limit evolution back to self-supporting growth forms”. Based on the evidence presented above we argue that evolutionary changes between arborescent and lianescent habits can be reversible, and that their frequency and trends seem to vary between groups. In addition, the weak-stem condition of shrubs and treelets in *Appunia* (observations by T. D. McDowell) and the scandent- or vining-branch condition of shrubs or treelets in the Neotropical *Morinda royoc* [26] may be viewed as a reflection of their origins from lianescent forms. We find no evidence of any reversal from arborescent to lianescent habit in the 61 Morindeae species included in this study. On the other hand, the sole lianescent species (*A. megalantha*) in the otherwise arborescent *Appunia*, not investigated in this study due to lack of material for sequencing, may represent a unique case of an evolutionary reversal from arborescent to lianescent habit in the tribe.

Furthermore, the acquisition of arborescent habit in *Appunia* seems to have coincided with the diversification of the genus in the Neotropics. Consequently, the evolutionary changes from lianescent to arborescent habit within Morindeae may in part be attributed to reduced competition for open ground and a scarcity of host trees for climbing plants in more open habitats [15]–[16]. This could explain the abundance of some species of the Asian, arborescent *Morinda* in sparse forests on hill slopes or open disturbed forests and the common occurrence of many Asian, lianescent *Gynochthodes* in forests or thickets on mountains [27]. The pantropical, arborescent *Morinda citrifolia* L. is also commonly found on seashores and sparse forests throughout its geographic ranges [26]–[27]. Similarly, the Neotropical *M. royoc* is common in pine savannas and coastal strands [26]. Finally, five *Appunia* species of the Neotropical Guianas region are shrubs, which frequently occur at forest edges, in clearings along riverbanks, and in disturbed, opened sites (observations by T. D. McDowell).

### Evolution of Inflorescence Architecture and its Evolutionary Implications

The majority of species in Morindeae with head inflorescence belong to *Gynochthodes*, although they occur in all five recognized genera (Table 1). Conversely, nine of the 11 *Coelospermum* species and 18 *Gynochthodes* species have non-headed inflorescences. If the weakly inferred head inflorescence at node A (Morindeae) is correct, this state is interpreted as plesiomorphic within Morindeae (for nodes B–G); this is inconsistent with our hypothesis of a derived head inflorescence within the tribe. The inferred plesiomorphic head inflorescence for nodes B–G, although weakly supported for nodes B–C and F–G, is consistent with highly to moderately supported plesiomorphic multiple fruits in nodes B–C and F–G. Multiple fruits can only be produced by taxa with head inflorescences, although plants with headed inflorescences can also produce simple fruits (e.g., *Appunia* (node D), Fig. 2). The evolutionary changes from headed to non-headed inflorescence occurred at least four times within Morindeae: twice each in *Coelospermum* (the Australian *C. reticulatum* and the New Caledonian *C. balansanum* group, Fig. 2) and *Gynochthodes* (the *G. coriacea* group and the Australian *G. retropila* (Halford & A.J.Ford) Razafim. & B.Bremer, Fig. 2). This is, to our current knowledge, the first report of evolutionary changes from headed to non-headed inflorescences in Rubiaceae.

The findings of this study raise new interesting questions. We do not know if the formation of non-headed from head inflorescences passes through the development of pedicels (umbels) followed by the formation of inflorescence branches in the umbellate forms to produce elongated, branched inflorescences. Alternatively, the non-headed inflorescence could be derived from a branched inflorescence of many heads if flower number was reduced to leave only one flower per receptacle. Unfortunately, discrete state ASR cannot tell us anything about the intermediate evolutionary changes leading to the formation of non-headed inflorescences in *Coelospermum* and *Gynochthodes*. Detailed comparative morphological and developmental studies combined with phylogeny are essential in order to elucidate the underlying developmental basis between the states of inflorescence architecture in Morindeae [28]–[29].

### Evolution of Flower Size in Morindeae and its Ecological and Evolutionary Implications

Almost all species of Morindeae with large flowers belong to the arborescent *Appunia* and *Morinda*, with the exception of the sole lianescent *Appunia* species, *A. megalantha* [12], and the two lianescent *Morinda* species, *M. longiflora* and *M. morindoides*. Conversely, Morindeae plants with small flowers are mostly the lianescent species of *Coelospermum*, *Gynochthodes*, and *Siphonandrium*, except the two arborescent *Gynochthodes* species (*G. decipiens* and *G. trimera*) and the lianescent *G. sublanceolata* Miq. Our ASRs strongly infer large flowers at the Morindeae-Mitchellae root node as well as node A (Morindeae), meaning that this state is plesiomorphic for the tribe Morindeae, the *Morinda-Coelospermum*-*Gynochthodes* clade (node B), *Appunia* (node D), and *Morinda* (node E). Small flowers are derived for the *Coelospemum-Gynochthodes* clade (node C), *Coelospermum* (node F), and *Gynochthodes* (node G). In other words, small flowers seem to have evolved only once from the large flowers within Morindeae (Fig. 2). It is worth noting that *G. sublanceolata* and *G. decipiens*, with large flowers but not included in this study, may represent one or two cases of reversals from small to large flowers.

The *Coelospemum-Gynochthodes* clade (node C) contains over 60% of the species in the tribe, and produce small flowers with inconspicuous colors that are most likely to be pollinated by small insects (e.g., short-proboscis moths or small bees or flies). Pollinators with progressively shorter proboscis may have been driving the transition from large to small flowers and an accompanying increase in speciation rate. Furthermore, change from large to small flowers in the *Coelospemum-Gynochthodes* clade appears to have been associated with a gender dimorphism transition. Androgynoecious (male and hermarphroditic) and dioecious conditions are only known from the lianescent species of *Gynochthodes* and *Coelospermum* [19]–[24] (Table 1). Thus, the high incidence of dioecy in the *Coelospermum-Gynochthodes* clade is correlated with woody, climbing growth habit, small flowers pollinated probably by unspecialized pollinators, and fleshy fruits. This pattern is consistent with those that have been reported from island habitats and various tropical forests [30]–[37]. Therefore, this study presents further support for the importance of these traits in the evolution of dioecy. On the other hand, it is important to note that all hermaphroditic members of *Appunia* and *Morinda* with large flowers also have the woody (but arborescent or frutescent) habit and fleshy fruits. This suggests that woodiness and fruit fleshiness alone cannot fully predict dioecy in the tribe Morindeae. In sum, the members of the *Coelospemum-Gynochthodes* clade display island syndrome characteristics, which are consistent with the fact that many of their species are indeed island endemics [13], [24].

In contrast to the *Coelospermum-Gynochthodes* clade, the large, mostly white flowers of *Appunia* and *Morinda* may be pollinated by larger insects, such as long-proboscis moths. This is consistent with the report on the Asian, arborescent *Morinda coreia* Buch.-Ham. being pollinated by hawkmoths in India [18]. The fact that the species of *Appunia* and *Morinda* are hermarphroditic suggests a single origin of dioecy in the *Coelospermum-Gynochthodes* clade from hermaphroditism. Members of *Appunia* and *Morinda* are predominantly distributed in continental areas (Africa mainland, continental Asia, and South and Central America), and show characteristics of the mainland pollinations and floral traits [30], [33]–[34], [36]–[38].

### Evolution of Multiple Fruits in Morindeae and its Ecological and Evolutionary Implications

Most Morindeae, about 90% of the species, bear multiple fruits. The majority of these species belong to *Gynochthodes* and *Morinda*, with only three species (two investigated in this study, Fig. 2) in *Coelospermum*. The infructescences of *Appunia* and *Siphonandrium* are composed of simple, drupaceous fruits. Our ASRs with moderate certainty infer multiple fruits at node Morindeae-Mitchelleae and node A (Morindeae). If correct, this state is plesiomorphic for Morindeae, the *Coelospermum-Gynochthodes* clade (node C), *Morinda* (node E), *Coelospermum* (node F), and *Gynochthodes* (node G) (Tables 2,3). This is inconsistent with the hypothesis of a derived multiple fruit for the broadly delimited *Morinda* (including the lianescent *Morinda* species transferred to *Gynochthodes* sensu Razafimandimbison et al. [8]–[9]), as postulated by McClatchey [11]. Simple, drupaceous fruits are derived for *Appunia* (node D) and seem to have arisen at least five times within Morindeae: once in *Appunia*, twice each in *Coelospermum* (the Australian *C. reticulatum* and the New Caledonian *C. balansanum* group), and *Gynochthodes* (the *G. coriacea* group and the Australian *G. retrophila*) (Fig. 2). This is, to our knowledge, the first report of an evolutionary transition from multiple to simple fruits in Rubiaceae. Within the *Coelospermum-Gynochthodes* clade (node C) the evolutionary change from multiple to simple fruits coincides with that of from headed to non-headed inflorescences. However, it is interesting that *Appunia* (node D) seems to have retained the plesiomorphic headed inflorescences but acquire simple fruits.

Like the acquisition of arborescent habit, the derivation of simple fruits in *Appunia* seems to have coincided with the divergence of the *Appunia* lineage in the Neotropics. The change from multiple to simple fruits in this genus is in part attributed to shifts in seed dispersal vectors. Seeds of the simple, drupaceous fruits of *Appunia* species are presumably dispersed by birds, whereas seeds of the larger multiple fruits are dispersed effectively by large frugivorous animals [3]. The same mechanism seems to underlie the evolutionary change from multiple to simple fruits within the *Coelospemum-Gynochthodes* clade.

The degree of ovary fusion prior to fruit development in head inflorescences varies greatly from only a basal, partial fusion to completely fused ovaries among *Morinda* and *Gynochthodes*. This variation, which is rarely mentioned by Rubiaceae systematists [26], merits consideration for its ecological implications. Clusters of simple fruits of *Appunia* are likely to be dispersed individually by frugivorous birds. Multiple fruits composed of partly to fully fused ovaries are presumably dispersed as single units, while those formed by basally fused ovaries could well be dispersed individually by frugivorous birds or as single units by larger frugivorous dispersers. Furthermore, we suspect that in many members of *Gynochthodes* and *Morinda* ovaries of the adjacent flowers are basally fused prior to and during maturation of the anthers, and that ovary fusion extends midway during fructification. This type of ovary fusion was reported for *Breonia richardsonii* Razafim. in the tribe Naucleeae of the subfamily Cinchonoideae (Rubiaceae) by Razafimandimbison [39].

### Future Perspectives

The Bayesian phylogenetic approach used here provides a sound framework for examining the evolution of distinctive vegetative (growth habit) and reproductive traits (flower, inflorescence, and fruit structures), which have broad ecological importance and potential impact on our understanding of speciation and diversity. Methods, which rely upon mapping discrete character states across a phylogeny, inevitably reduce the complexity of character variation among a diverse group of species. Thus, the arborescent habit includes all non-liana woody shrubs and trees (large trunked trees (e.g., noni, *Morinda citrifolia*), suffrutescent plants (e.g., *M. buchii* Urb.), shrubs or treelets with scandent- or vining-branches (e.g., *Morinda royoc*), and weakly branching treelets (e.g., *Appunia debilis* Sandwith)). Similarly, the character states "large flower" and "small flower" and their diagnosis based upon corolla tube/lobe ratio summarize diverse flower sizes. The presence or absence of head inflorescences involves the complication of comparing much-branched inflorescences with unbranched inflorescences: either may have flowers in heads or not. Fruit fusion, though variable in degree, is summarized in the character states as simple or multiple. Despite the simplification of diverse characteristics into discrete character states, the essential outcomes of these analyses are clearly evident across the phylogenetic span of this inquiry: repeated shifts have occurred in the evolution of the growth habit, inflorescence architecture, flower size, and fruit across the species of the Morindeae. Moreover, the direction of these evolutionary changes has often been unexpected and at odds with currently accepted hypotheses. Finally, the findings of this study provide a new context for viewing patterns of character evolution and examining their ecological and developmental basis.

## Materials and Methods

### Taxon Sampling and Data Collection

The sampling used for this study coincided with the molecular phylogenetic study of Morindeae by Razafimandimbison et al. [9], on the basis of which new generic limits of the tribe were established. This latter study resulted in the transfer of all lianescent, dioecious *Morinda* species to *Gynochthodes* and all species of *Sarcopygme* Setch & Christoph. to *Morinda* [8]–[9]. Accordingly, the newly combined names of *Morinda* and *Gynochthodes*, respectively, were utilized in this study to replace the names of the sampled *Sarcopygme* and lianescent *Morinda* used in Razafimandimbison et al. [9]. Five Morindeae taxa (*Appunia tenuiflora* (Benth.) Jacks & Hook.f., *Morinda royoc* L. 2, and *Gynochthodes candollei* Montrouz. 2, 4, and 5) with incomplete sequences were excluded from this study to decrease the percentage of missing information in the combined nrETS/nrITS/*trn*T-F matrix and obtain a well-resolved phylogeny of Morindeae for basing our ASRs. We investigated a total of 66 taxa, and all information about the voucher specimens and sequences used in the study is published in Razafimandimbison et al. [9].

All morphological characteristics of the five genera of Morindeae summarized in Table 1 were based on data from field notes made by SGR (for *Coelospermum*, *Gynochthodes*, and the paleotropical *Morinda*) and by TDM (for *Appunia* and the Neotropical *Morinda*). This was coupled with data compiled by SGR from herbarium specimens on loan from many herbaria (BR, K, L, MO, P, S, TAN, TEF, UPS, [40]) and the literature [8]–[9], [12]–[13], [21]–[22], [24], [26]–[27].

### Laboratory Work and Phylogenetic Analyses

The protocols used for DNA extraction, amplification, and sequencing are outlined in Razafimandimbison et al. [9]. The alignment of the combined nrETS/nrITS/*trn*T-F data was re-adjusted after the removal of *A. tenuiflora*, *M. royoc* 2, and *G. candollei* 2, 4, and 5. We treated each of the three gene regions as a separate partition and selected likelihood models following Razafimandimbison et al. [9]. As a consequence, we applied separately parameterized GTR+ G models to the *trn*T-F and nrITS partitions and a separately parameterized HKY+ G model to the nrETS partition. The gamma distributed rate heterogeneity across sites was approximated with four discrete categories. Flat Dirichlet priors were applied to the state frequencies and to the substitution rates of the GTR model, whereas a flat beta distribution was used as prior for the transition-to-transversion rate. A uniform prior on the interval (0.1, 50) was applied to the gamma curve shape parameter α. The prior on branch lengths was an exponential distribution with mean 0.1. Rate heterogeneity across partitions was modeled according to a proportional model with a flat Dirichlet prior. Tree topologies were treated as a priori equally likely. Three runs of Metropolis-coupled MCMC was run for 25×10^6^ generations, each run starting from a random tree with initial branch lengths set to 0.1. Each run included four chains, three of which were incrementally heated to a temperature of 0.15 to ensure swap rates between adjacent chains between 10 and 70%. Every 1000^th^ generation of the cold MCMC chain was sampled. Stationarity and convergence of runs, as well as the correlation of split frequencies between the runs were checked using the program AWTY [41]. We checked the effective sample size (ESS) of parameters using the program Tracer v.1.5.0 [42]. Trees sampled from the first 12.5×10^6^ generations were discarded as burn-in. All saved trees (after excluding burn-ins) from the three independent runs were pooled for a consensus tree.

### Reconstruction of Ancestral States

A variety of comparative phylogenetic methods have been used for reconstructing ancestral states of characters and mapping character changes across lineages: maximum parsimony [43]–[44], maximum likelihood [45], Bayesian inference [46]–[48], and stochastic character mapping [49]. The influences of method choice in reconstructing ancestral states of characters are well documented [2], [50]; it has recently been demonstrated that homoplasious characters are sensitive to choice of method [2].

The Bayesian approach implemented in the computer program BayesTraits v. 1.0 [48] appears to preserve the highest amount of uncertainty in ASR of discrete characters [2], [50]. It takes into account both phylogenetic uncertainty and branch length, and also permits one to explore a variety of models for character transition and to investigate nodes of interest [2], [50]. We performed ASRs of the four characters of the tribe Morindeae (growth habit, inflorescence architecture, flower size, and fruit type) using the software BayesTraits as described by [48] and on two posterior tree samples, one in which all outgroup taxa (Fig. 2) had been pruned and one in which we kept the outgroup taxa *Mitchella repens* and *Damnacanthus indicus* of the tribe Mitchelleae, known from previous studies to be the closest relatives of the Morindeae [51]. *Pagamea guianensis* Aubl. and *Gaertnera phyllostachya* Baker were pruned from the analyses, because they represent the poorly sampled tribe Gaertnereae and appear on long branches in the phylogeny. Before proceeding with the ASR, we checked trees for the node-density artifact [52] using the on-line implementation at http://www.evolution.reading.ac.uk/pe/index.html. The following four discrete characters were reconstructed for the ingroup taxa: growth form lianescent (0), arborescent (including frutescent and suffrutescent plants of *Morinda*, the weak-stemmed shrubs or treelets of *Appunia*, and the scandent- or vining branched shrubs or treelets of *Morinda royoc* L., i.e., all non-liana woody shrubs and trees) (1), and herbaceous (only relevant for the outgroup taxon *M. repens*) (2); inflorescence headed (0) or non-headed (1); flowers large (corolla tube length/corolla lobe length >1) (0) or small (corolla tube length/corolla lobe length <1) (1); and fruits simple (0) or fused (1). State probabilities were estimated for the following seven selected nodes in the Bayesian majority rule consensus tree (Fig. 2): Morindeae (node A), the *Morinda*-*Coelospermum*-*Gynochthodes* clade (node B), the *Coelospermum*-*Gynochthodes* clade (node C), *Appunia* (node D), *Morinda* (node E), *Coelospermum* (node F), and *Gynochthodes* (node G). Node A corresponds to the root of the tree when the outgroup taxa had been excluded. In addition, we reconstructed the root node (joining the Mitchelleae and Morindeae) in the analyses involving the two outgroup taxa of Mitchelleae. Reversible-jump MCMC was used to integrate over models. For single binary characters, there are four possible models, one two-rate model in which forward (q_01_) and backward (q_10_) rates are free, one single-rate model in which q_01_ and q_10_ are constrained to be equal, two single-rate models in which either q_01_ or q_10_ is estimated, and the reverse rate is fixed to zero. Ratios of q_01_ to q_10_ deviating from 1 indicate that the rate of change in one direction is higher than in the opposite direction.

We used a uniform prior on the models and an exponential prior on rates, the mean of which was seeded by a uniform hyperprior on the interval (0, 10). By applying an exponential prior on rates we say that moderate rates are a priori more likely than high rates and that strong evidence from the data is required to accept high-rate estimates. We also included the branch-length transformation parameter κ in the model [53]. This parameter raises original branch lengths to the κ power. If κ  = 0, all branches are equally long, i.e., change is independent of branch lengths. If κ  = 1, branch lengths are not modified and change is perfectly proportional to the original branch lengths. κ >1 indicates that change accelerates with increasing branch length and 0< κ <1 indicates that change decelerates with decreasing branch lengths. The prior on κ is a uniform distribution on the interval (0, 5) (A. Meade, pers. com.). The MCMC was run for 220×10^6^ generations, the first 20×10^6^ of which were discarded as burnin. A sample was saved from the posterior every 1000th generation. The rate deviation of the normal distribution was set to obtain an MCMC acceptance rate between 20% and 40%. Each analysis was conducted three times to check that similar harmonic mean likelihoods were obtained across runs.
